# Supplementary material for: Breast Cancer Brain Metastasis—Overview of Disease State, Treatment Options and Future Perspectives
Source: Cancers (Basel). 2021 Mar 3;13(5):1078. doi: 10.3390/cancers13051078 (PMC7959316; doi:10.3390/cancers13051078)
Supplement: Supplementary file 1 [file cancers-13-01078-s001.pdf]

Table S1. Clinical trials in local treatment for BCBM

Local treatment for BCBM						
**Treatment**	**Patients' population**	**Trial name (NCT number)**	**Phase**	**Estimated primary completion date**	**Status**	**Primary endpoint**
Preoperative SRS vs Postoperative SRS	BMs under 4 cm for single fraction, and over 7 cm for multifraction therapy	(NCT03741673)	III	July 10, 2022	Recruiting	LMD-free rate
Preoperative SRS	1–6 BMs (2–4cm)	(NCT03368625)	II	January 1, 2022	Recruiting	Radiation toxicity
WBRT vs HA-WBRT	New BM which at least 2 BMs are under 40 mmm, and the distance from the border of a mass to the hippocampal margin should be more than 15 mm.	(NCT03002532)	(Not Applicable)	August, 2017	Unknown	Neurocognitive function
SRS with WBRT versus SRS without WBRT	5–20 BMs	(NCT03775330)	Not applicable	December, 2020	Not yet recruiting	Neurocognitive function
WBRT with memantine vs WBRT without memantine	Pathologically proven BMs	(NCT00566852)	III	April, 2011	Published by Brown et al. [1]	Cognitive function
WBRT vs HA-WBRT	New BM which at least 2 BMs are under 40 mmm, and the distance from the border of a mass to the hippocampal margin should be more than 15 mm.	(NCT03002532)	(Not Applicable)	August, 2017	Unknown	Neurocognitive function
HA-WBRT plus memantine vs WBRT plus memantine	BMs outside a 5-mm margin around either hippocampus	NRG Oncology CC001 (NCT02360215)	III	April 30, 2018	Published by Brown et al. [2]	Time to cognitive function failure
SRS vs HA-WBRT plus memantine	5–15 BMs	(NCT03550391)	III	December 31, 2021	Recruiting	OS, Neurocognitive PFS

FSRT	1 to 10 HER2-positive BCBMs	(NCT04061408)	II	July, 2021	Recruiting	Intracranial local tumor control rate

Abbreviations: BM, brain metastasis; BCBM, breast cancer brain metastasis; SRS, stereotactic radio surgery; WBRT, whole brain radiation therapy; HA-WBRT, hippocampal-avoidance whole brain radiation therapy; FSRT, fractionated stereotactic radiotherapy; OS, overall survival; PFS, progression free survival; LMD, leptomeningeal disease

Table S2. Clinical trials in systemic therapy for BCBM

**Systemic therapy for HER2-positive BCBM**						
**Treatment**	**Patients' population**	**Trial name (NCT number)**	**Phase**	**Estimated primary completion date**	**Status**	**Primary endpoint**
Intra-arterial cerebral infusion of trastuzumab	HER2-positive BCBM	(NCT02571530)	I	September, 2022	Recruiting	MTD, adverse events and dose-limiting toxicities
Lapatinib plus capecitabine vs capecitabine	Advanced BC with progression on trastuzumab	(NCT00078572)	III	April, 2006	Published by Cameron et al. [3]	Time to progression
Lapatinib plus capecitabine vs lapatinib plus topotecan	HER2-positive BC with progressive BCBM after trastuzumab and cranial radiotherapy	(NCT00437073)	II	January, 2009	Published by Lin et al. [4]	CNS objective response
Lapatinib plus taxane followed by lapatinib vs trastuzumab plus taxane followed by trastuzumab	HER2-positive MBC without BM	NCIC CTG MA.31 (NCT00667251)	III	August 1, 2012	Published by Gelmon et al. [5]	PFS
Lapatinib plus capecitabine vs trastuzumab plus capecitabine	HER2-positive MBC without BM	CEREBEL (EGF111438) (NCT00820222)	III	June 11, 2012	Published by Pivot et al. [6]	Incidence of CNS metastases as first site of relapse
Intermittent high-dose Lapatinib plus capecitabine	HER2-positive BCBM	(NCT02650752)	I	January 22, 2021	Published by Morikawa et al.[7]	MTD
Lapatinib plus everolimus plus capecitabine	HER2-positive BCBM	TRIO-US B-09 (NCT01783756)	I/II	May 10, 2019	Published by Hurvitz et al. [8].	CNS ORR
Lapatinib plus WBRT/SRS vs WBRT/SRS	HER2-positive BCBM	(NCT01622868)	II	December 30, 2019	Reported by Kim et al.[9]	CR rate in the measurable BM
Lapatinib plus WBRT	BM from HER2-positive BC or lung cancer	(NCT01218529)	II	August, 2014	Completed	Response rate in brain as assessed by volumetric analysis of brain MRI.
Pertuzumab plus high-dose trastuzumab	HER2-positive progressive BCBM after radiotherapy	PATRICIA (NCT02536339)	II	May 1, 2019	Ongoing	Percentage of participants with objective response in the CNS

Intrathecal pertuzumab and trastuzumab	New untreated asymptomatic or low symptomatic HER2-positive BCBM	(NCT02598427)	I	February 22, 2018	Terminated (Inadequate enrollment)	Safety, MTD
T-DM1 alone vs T-DM1 plus metronomic temozolomide	HER2-positive BCBM treated with SRS	(NCT03190967)	I/II	June 30, 2022	Recruiting	MTD of temozolomide when used with T-DM1, Median amount of time subject survives without disease progression after treatment
Afatinib vs afatinib plus 2Gy radiotherapy vs afatinib plus 4Gy radiotherapy	BM from BC or lung cancer	(NCT02768337)	I/II	June, 2021	Recruiting	Ratio of afatinib concentration in resected BM / plasma
Tucatinib plus T-DM1	HER2-positive MBC	(NCT01983501)	I	October 10, 2017	Published by Borges et al. [10]	Incidence of adverse events
Neratinib	Stage II to IIIC HER-2 positive breast cancer with node positive disease	ExteNET (NCT00878709)	III	August 21, 2014	Published by Chan et al. [11]	Invasive disease-free survival at year 2
Pyrotinib plus vinorelbine	HER2-positive BCBM	(NCT03933982)	II	December 22, 2021	Recruiting	ORR of CNS
Pyrotinib plus capecitabine	HER2-positive BCBM	(NCT03691051)	II	November 20, 2019	Unknown	ORR of CNS
Pyrotinib plus temozolomide (for HER2-positive BCBM), SHR1316 and bevacizumab plus cisplatin or carboplatin (for TN type BCBM)	HER2-positive or TN-type BCBM	(NCT04303988)	II	December 30, 2021	Not yet recruiting	ORR in CNS
GRN1005 (paclitaxel trevatide) vs GRN1005 plus trastuzumab	HER2-positive BCBM	GRABM-B (NCT01480583)	II	June, 2015	Completed and reported by Bates et al. [12]	ORR in CNS
GDC-0084 plus trastuzumab	HER2-positive BCBM	(NCT03765983)	II	November30, 2021	Recruiting	Overall response rate in the CNS, and

						evaluation of the correlation between inhibition of p-4EBP1 in resected BM and intracranial response in the corresponding PDX models of BCBM
Palbociclib plus trastuzumab	HR-negative/HER2-positive BCBM	(NCT02774681)	II	February 13. 2019	Terminated (Slow accrual)	RRR in CNS
Palbociclib plus trastuzumab plus lapatinib plus fulvestrant	ER-positive/HER2-positive BCBM	(NCT04334330)	II	July, 2021	Not yet recruiting	ORR in CNS
Everolimus plus vinorelbine plus trastuzumab	HER2-positive BCBM	(NCT01305941)	II	August, 2016	Published by Van Swearingen et al.[13]	ORR in CNS
HER2-CAR T cells	HER2-positive recurrent BCBM or leptomeningeal metastases	(NCT03696030)	I	August 31, 2021	Recruiting	Incidence of DLTs, number of adverse events
HER2-specific T cells (HER2-CAR T cells)	HER2-positive BCBM	(NCT02442297)	I	February, 2021	Recruiting	Number of patients with DLTs
Atezolizumab plus pertuzumab plus trastuzumab	HER2-positive BCBM	(NCT03417544)	II	February, 2021	Active, not recruiting	ORR in CNS
**Systemic therapy for luminal-type BCBM**						
**Treatment**	**Patients' population**	**Trial name (NCT number)**	**Phase**	**Estimated primary completion date**	**Status**	**Primary endpoint**
Everolimus plus exemestane vs everolimus alone vs capecitabine alone	HR-positive locally advanced, recurrent or MBC	BOLERO-6 (NCT01783444)	II	July 2, 2018	Published by Jerusalem et al. [14]	PFS
Abemaciclib or PI3K inhibitor GDC-0084 or entrectinib, selected by genetic test	New or progressive BM	(NCT03994796)	II	July, 2021	Recruiting	ORR in CNS
Ribociclib (LEE011) plus buparlisib (BKM120) plus fulvestrant vs ribociclib	Postmenopausal women with HR-positive/HER2-negative,	(NCT02088684)	I/II	April 17, 2018	Completed and reported by Tolaney et al. [15]	Incidence of DLTs, PFS

plus alpelisib (BYL719) plus fulvestrant vs ribociclib plus fulvestrant	locally recurrent or advanced MBC					
Buparlisib (BKM120) plus fulvestrant vs fulvestrant	HR-positive / HER2-negative, locally advanced or MBC, with postmenopausal status	BELLE-2 (NCT01610284)	III	April 20, 2015	Published by Baselga et al. [16]	PFS
Buparlisib (BKM120) plus capecitabine (plus trastuzumab for HER2 positive group)	BCBM (including all subtype)	(NCT02000882)	II	March 29, 2019	Completed	CBR
Alpelisib (BYL719) plus capecitabine vs buparlisib (BKM120) plus capecitabine vs buparlisib plus capecitabine plus trastuzumab vs buparlisib plus capecitabine plus lapatinib	MBC including BCBM	(NCT01300962)	I	February 7, 2017	Published by McRee et al. [17]	MTD, DLT
**Systemic therapy for triple negative BCBM**						
**Treatment**		**Trial name (NCT number)**	**Phase**	**Estimated primary completion date**	**Status**	**Primary endpoint**
Pembrolizumab plus SRS	BCBM (including all subtype)	(NCT03449238)	I/II	December 30, 2024	Recruiting	Tumor response for non-irradiated brain lesions at 8 weeks, correlation of abscopal responses with the radiation dose received, OS
Pembrolizumab vs chemotherapy (treatment of physician's choice)	Metastatic TNBC	NK-3475–119/KEYNOTE-119 (NCT02555657)	III	April 11, 2019	Completed	OS
Pembrolizumab plus paclitaxel vs pembrolizumab plus nab-paclitaxel vs pembrolizumab plus	Previously untreated locally recurrent inoperable BC or metastatic TNBC	MK-3475-355/KEYNOTE-355 (NCT02819518)	III	January 12, 2022	Active, not recruiting. Reported by Cortes et al. [18]	Adverse event, PFS, OS

gemcitabine/carboplatin vs pembrolizumab plus chemotherapy vs chemotherapy						
Atezolizumab plus SRS	TN-type BCBM	(NCT03483012)	II	September 30, 2021	Active, not recruiting	PFS
Atezolizumab plus paclitaxel vs paclitaxel alone	Previously untreated locally advanced or metastatic TNBC	IMpassion131 (NCT03125902)	III	November 15, 2019	Active, not recruiting<br>Reported by Miles et al. [19]	PFS
Atezolizumab plus chemotherapy (pegylated liposomal doxorubicin plus cyclophosphamide) vs chemotherapy	Locally advanced, or metastatic TNBC	ALICE (NCT03164993)	II	August 1, 2021	Recruiting	Toxicity, PFS
Nivolumab plus SRS	BCBM (including all subtype)	(NCT03807765)	I	September 8, 2020	Active, not recruiting	Number of participants who experience DLTs
Neoadjuvant nivolumab plus ipilimumab vs none prior to surgery	1 to 3 untreated, at least surgically-resectable, solid tumor BM	(NCT04434560)	II	January, 2023	Recruiting	Proportion of patients that receive neoadjuvant ipilimumab and nivolumab prior to surgery and SRS, proliferation of circulating T-cells
Sorafenib plus WBRT	BCBM (including all subtype)	(NCT01724606)	I	November, 2021	Active, not recruiting	MTD, toxicity by the number of adverse events
AZD1390 plus radiation therapy	Primary brain tumor or BM	(NCT03423628)	I	February 17, 2023	Recruiting	Incidence of DLTs, incidence of adverse events and serious adverse events
Etirinotecan pegol (NKTR-102) vs physician's treatment of choice	Locally recurrent BC or MBC previously treated with an anthracycline, a taxane and capecitabine	BEACON (NCT01492101)	III	April, 2016	Published by Cortés et al. [20]	OS
Erinotecan pegol (NKTR-102) vs physician's treatment of choice	Stable BCBM previously treated with an anthracycline, a taxane and capecitabine	ATTAIN (NCT02915744)	III	July, 2020	Published by Tripathy et al. [21]	OS
Nanoliposomal irinotecan	BCBM	(NCT03328884)	II	October 15,	Recruiting	ORR in CNS

	(including all subtype)			2022		
Veliparib (ABT-888) plus carboplatin plus paclitaxel vs carboplatin plus paclitaxel	HER2-negative metastatic or locally advanced unresectable BC with *BRCA* mutation	BROCADE3 (NCT02163694)	III	April 5, 2019	Published by Diéras et al. [22]	PFS
Cisplatin plus veliparib vs cisplatin alone	Recurrent or Metastatic TNBC, including BM	SWOG S1416 (NCT02595905)	II	October 31, 2021	Active, not recruiting<br>Reported by Sharma et al. [23]	PFS
Talazoparib (BMN-673) plus carboplatin plus paclitaxel	Advanced *BRCA*-mutated solid tumor or metastatic TNBC	(NCT02358200)	I	January 8, 2019	Terminated	ORR
Rucaparib	HER2-negative MBC with a BRCAness	RUBY (NCT02505048)	II	February, 2019	Active, not recruiting<br>Reported by Patsouris et al. [24]	CBR
Niraparib plus pembrolizumab	Advanced or metastatic TNBC or recurrent ovarian cancer	TOPACIO/KEYNOTE-162 (NCT02657889)	I/II	May 14, 2018	Published by Vinayak et al. [25]	Number of subjects reporting DLTs, ORR

Abbreviations: HR, hormone receptor; HER2, human epidermal growth factor receptor 2; TN, triple negative; TNBC, triple negative breast cancer; BC, breast cancer; BM, brain metastasis; BCBM, breast cancer brain metastasis; MBC, metastatic breast cancer; SRS, stereotactic radiosurgery; CNS, central nervous system; CAR, Chimeric Antigen Receptors; CSF, cerebrospinal fluid; OS, overall survival; PFS, progression-free survival; ORR, objective response rate; RRR, radiographic response rate; CBR, clinical benefit rate; CR, complete response; T-DM1, trastuzumab emtansine; MRI, magnetic resonance imaging; PDX, patient-derived xenograft; MTD, maximum tolerated dose; DLTs, dose limiting toxicities

References

1. Brown, P.D.; Pugh, S.; Laack, N.N.; Wefel, J.S.; Khuntia, D.; Meyers, C.; Choucair, A.; Fox, S.; Suh, J.H.; Roberge, D.; et al. Memantine for the prevention of cognitive dysfunction in patients receiving whole-brain radiotherapy: A randomized, double-blind, placebo-controlled trial. *Neuro. Oncol.* **2013**, *15*, 1429–1437, doi:10.1093/neuonc/not114.
2. Brown, P.D.; Gondi, V.; Pugh, S.; Tome, W.A.; Wefel, J.S.; Armstrong, T.S.; Bovi, J.A.; Robinson, C.; Konski, A.; Khuntia, D.; et al. Hippocampal Avoidance During Whole-Brain Radiotherapy Plus Memantine for Patients With Brain Metastases: Phase III Trial NRG Oncology CC001. *J. Clin. Oncol.* **2020**, *38*, 1019–1029, doi:10.1200/jco.19.02767.
3. Cameron, D.; Casey, M.; Press, M.; Lindquist, D.; Pienkowski, T.; Romieu, C.G.; Chan, S.; Jagiello-Gruszfeld, A.; Kaufman, B.; Crown, J.; et al. A phase III randomized comparison of lapatinib plus capecitabine versus capecitabine alone in women with advanced breast cancer that has progressed on trastuzumab: Updated efficacy and biomarker analyses. *Breast Cancer Res. Treat.* **2008**, *112*, 533–543, doi:10.1007/s10549-007-9885-0.
4. Lin, N.U.; Eierman, W.; Greil, R.; Campone, M.; Kaufman, B.; Steplewski, K.; Lane, S.R.; Zembryki, D.; Rubin, S.D.; Winer, E.P. Randomized phase II study of lapatinib plus capecitabine or lapatinib plus topotecan for patients with HER2-positive breast cancer brain metastases. *J. Neurooncol.* **2011**, *105*, 613–620, doi:10.1007/s11060-011-0629-y.
5. Gelmon, K.A.; Boyle, F.M.; Kaufman, B.; Huntsman, D.G.; Manikhas, A.; Di Leo, A.; Martin, M.; Schwartzberg, L.S.; Lemieux, J.; Aparicio, S.; et al. Lapatinib or Trastuzumab Plus Taxane Therapy for Human Epidermal Growth Factor Receptor 2–Positive Advanced Breast Cancer: Final Results of NCIC CTG MA.31. *J. Clin. Oncol.* **2015**, *33*, 1574–1583, doi:10.1200/jco.2014.56.9590.
6. Pivot, X.; Manikhas, A.; Żurawski, B.; Chmielowska, E.; Karaszewska, B.; Allerton, R.; Chan, S.; Fabi, A.; Bidoli, P.; Gori, S.; et al. CEREBEL (EGF111438): A Phase III, Randomized, Open-Label Study of Lapatinib Plus Capecitabine Versus Trastuzumab Plus Capecitabine in Patients With Human Epidermal Growth Factor Receptor 2–Positive Metastatic Breast Cancer. *J. Clin. Oncol.* **2015**, *33*, 1564–1573, doi:10.1200/jco.2014.57.1794.
7. Morikawa, A.; De Stanchina, E.; Pentsova, E.; Kemeny, M.M.; Li, B.T.; Tang, K.; Patil, S.; Fleisher, M.; Van Poznak, C.; Norton, L.; et al. Phase I Study of Intermittent High-Dose Lapatinib Alternating with Capecitabine for HER2-Positive Breast Cancer Patients with Central Nervous System Metastases. *Clin. Cancer Res.* **2019**, *25*, 3784–3792, doi:10.1158/1078-0432.ccr-18-3502.
8. Hurvitz, S.; Singh, R.; Adams, B.; Taguchi, J.A.; Chan, D.; Dichmann, R.A.; Castrellon, A.; Hu, E.; Berkowitz, J.; Mani, A.; et al. Phase Ib/II single-arm trial evaluating the combination of everolimus, lapatinib and capecitabine for the treatment of HER2-positive breast cancer with brain metastases (TRIO-US B-09). *Ther. Adv. Med. Oncol.* **2018**, *10*, 175883591880733, doi:10.1177/1758835918807339.
9. Kim, I.A.; Moughan, J.; Sperduto, P.W.; De Los Santos, J.F.; Peereboom, D.; Ogunleye, T.B.; Boulter, D.; Cho, K.H.; Shin, K.H.; Zoberi, I.; et al. NRG Oncology/RTOG 1119: PHASE II Randomized Study of Whole Brain Radiotherapy/Stereotactic Radiosurgery with Concurrent Lapatinib in Patients with Brain Metastases from HER2-Positive Breast Cancer—A Collaborative Study of NRG and KROG (NCT01622868). *International Journal of Radiation Oncology*Biology*Physics* **2020**, *108*, S174-S175, doi:10.1016/j.ijrobp.2020.07.953.
10. Borges, V.F.; Ferrario, C.; Aucoin, N.; Falkson, C.; Khan, Q.; Krop, I.; Welch, S.; Conlin, A.; Chaves, J.; Bedard, P.L.; et al. Tucatinib Combined With Ado-Trastuzumab Emtansine in Advanced ERBB2/HER2-Positive Metastatic Breast Cancer. *JAMA Oncology* **2018**, *4*, 1214, doi:10.1001/jamaoncol.2018.1812.
11. Chan, A.; Moy, B.; Mansi, J.; Ejlertsen, B.; Holmes, F.A.; Chia, S.; Iwata, H.; Gnant, M.; Loibl, S.; Barrios, C.H.; et al. Final Efficacy Results of Neratinib in HER2-positive Hormone Receptor-positive Early-stage Breast Cancer From the Phase III ExteNET Trial. *Clin. Breast Cancer* **2020**, 10.1016/j.clbc.2020.09.014, doi:10.1016/j.clbc.2020.09.014.
12. Bates, S.E.; Lindenberg, M.L.; Bryla, C.; Pichun, M.E.B.; Patronas, N.; Amiri-Kordestani, L.; Gonzalez, E.M.; Fojo, T.; Balasubramaniam, S.; Choyke, P.L. ANG1005 for brain metastases from breast cancer: 18F-FLT-PET and MRI as complementary approaches to response assessment. *J. Clin. Oncol.* **2015**, *33*, 2552–2552, doi:10.1200/jco.2015.33.15_suppl.2552.
13. Van Swearingen, A.E.D.; Siegel, M.B.; Deal, A.M.; Sambade, M.J.; Hoyle, A.; Hayes, D.N.; Jo, H.; Little, P.; Dees, E.C.; Muss, H.; et al. LCCC 1025: A phase II study of everolimus, trastuzumab, and vinorelbine to treat progressive HER2-positive breast cancer brain metastases. *Breast Cancer Res. Treat.* **2018**, *171*, 637–648, doi:10.1007/s10549-

018-4852-5.
14. Jerusalem, G.; De Boer, R.H.; Hurvitz, S.; Yardley, D.A.; Kovalenko, E.; Ejlertsen, B.; Blau, S.; Özgüroglu, M.; Landherr, L.; Ewertz, M.; et al. Everolimus Plus Exemestane vs Everolimus or Capecitabine Monotherapy for Estrogen Receptor–Positive, HER2-Negative Advanced Breast Cancer. *JAMA Oncology* **2018**, *4*, 1367, doi:10.1001/jamaoncol.2018.2262.
15. Tolaney, S.; Forero-Torres, A.; Boni, V.; Bachelot, T.; Lu, Y.-S.; Maur, M.; Fasolo, A.; Motta, M.; Pan, C.; Dobson, J.; et al. Abstract P4-22-12: Ribociclib + fulvestrant in postmenopausal women with HR+, HER2– advanced breast cancer (ABC). *Cancer Res.* **2017**, *77*, P4-22-12-P24-22-12, doi:10.1158/1538-7445.Sabcs16-p4-22-12.
16. Baselga, J.; Im, S.A.; Iwata, H.; Cortés, J.; De Laurentiis, M.; Jiang, Z.; Arteaga, C.L.; Jonat, W.; Clemons, M.; Ito, Y.; et al. Buparlisib plus fulvestrant versus placebo plus fulvestrant in postmenopausal, hormone receptor-positive, HER2-negative, advanced breast cancer (BELLE-2): A randomised, double-blind, placebo-controlled, phase 3 trial. *Lancet Oncol.* **2017**, *18*, 904–916, doi:10.1016/s1470-2045(17)30376-5.
17. McRee, A.J.; Marcom, P.K.; Moore, D.T.; Zamboni, W.C.; Kornblum, Z.A.; Hu, Z.; Phipps, R.; Anders, C.K.; Reeder-Hayes, K.; Carey, L.A.; et al. A Phase I Trial of the PI3K Inhibitor Buparlisib Combined With Capecitabine in Patients With Metastatic Breast Cancer. *Clin. Breast Cancer* **2018**, *18*, 289–297, doi:10.1016/j.clbc.2017.10.014.
18. Cortes, J.; Cescon, D.W.; Rugo, H.S.; Nowecki, Z.; Im, S.-A.; Yusof, M.M.; Gallardo, C.; Lipatov, O.; Barrios, C.H.; Holgado, E.; et al. KEYNOTE-355: Randomized, double-blind, phase III study of pembrolizumab + chemotherapy versus placebo + chemotherapy for previously untreated locally recurrent inoperable or metastatic triple-negative breast cancer. *J. Clin. Oncol.* **2020**, *38*, 1000, doi:10.1200/JCO.2020.38.15_suppl.1000.
19. Miles, D.; André, F.; Gligorov, J.; Verma, S.; Xu, B.; Cameron, D.; Barrios, C.; Schneeweiss, A.; Easton, V.; Ghazi, Y.; et al. Abstract OT1-01-01: IMpassion131: A phase III study comparing 1L atezolizumab with paclitaxel vs placebo with paclitaxel in treatment-naive patients with inoperable locally advanced or metastatic triple negative breast cancer (TNBC). *Cancer Res.* **2018**, *78*, OT1-01-01-OT01-01-01, doi:10.1158/1538-7445.Sabcs17-ot1-01-01.
20. Cortés, J.; Rugo, H.S.; Awada, A.; Twelves, C.; Perez, E.A.; Im, S.A.; Gómez-Pardo, P.; Schwartzberg, L.S.; Diéras, V.; Yardley, D.A.; et al. Prolonged survival in patients with breast cancer and a history of brain metastases: Results of a preplanned subgroup analysis from the randomized phase III BEACON trial. *Breast Cancer Res. Treat.* **2017**, *165*, 329–341, doi:10.1007/s10549-017-4304-7.
21. Tripathy, D.; Tolaney, S.M.; Seidman, A.D.; Anders, C.K.; Ibrahim, N.; Rugo, H.S.; Twelves, C.; Dieras, V.; Müller, V.; Tagliaferri, M.; et al. ATTAIN: Phase III study of etirinotecan pegol versus treatment of physician's choice in patients with metastatic breast cancer and brain metastases. *Future Oncology* **2019**, *15*, 2211–2225, doi:10.2217/fon-2019-0180.
22. Diéras, V.; Han, H.S.; Kaufman, B.; Wildiers, H.; Friedlander, M.; Ayoub, J.-P.; Puhalla, S.L.; Bondarenko, I.; Campone, M.; Jakobsen, E.H.; et al. Veliparib with carboplatin and paclitaxel in BRCA-mutated advanced breast cancer (BROCADE3): A randomised, double-blind, placebo-controlled, phase 3 trial. *The Lancet Oncology* **2020**, *21*, 1269–1282, doi:10.1016/s1470-2045(20)30447-2.
23. Sharma, P.; Rodler, E.; Barlow, W.E.; Gralow, J.; Huggins-Puhalla, S.L.; Anders, C.K.; Goldstein, L.J.; Brown-Glaberman, U.A.; Huynh, T.-T.; Szyarto, C.S.; et al. Results of a phase II randomized trial of cisplatin +/- veliparib in metastatic triple-negative breast cancer (TNBC) and/or germline BRCA-associated breast cancer (SWOG S1416). *J. Clin. Oncol.* **2020**, *38*, 1001–1001, doi:10.1200/JCO.2020.38.15_suppl.1001.
24. Patsouris, A.; Tredan, O.; Nenciu, D.; Tran-Dien, A.; Campion, L.; Goncalves, A.; Arnedos, M.; Sablin, M.-P.; Gouraud, W.; Jimenez, M.; et al. RUBY: A phase II study testing rucaparib in germline (g) BRCA wild-type patients presenting metastatic breast cancer (mBC) with homologous recombination deficiency (HRD). *J. Clin. Oncol.* **2019**, *37*, 1092–1092, doi:10.1200/JCO.2019.37.15_suppl.1092.
25. Vinayak, S.; Tolaney, S.M.; Schwartzberg, L.; Mita, M.; McCann, G.; Tan, A.R.; Wahner-Hendrickson, A.E.; Forero, A.; Anders, C.; Wulf, G.M.; et al. Open-label Clinical Trial of Niraparib Combined With Pembrolizumab for Treatment of Advanced or Metastatic Triple-Negative Breast Cancer. *JAMA Oncology* **2019**, *5*, 1132, doi:10.1001/jamaoncol.2019.1029.
